# Bilaterally originating double left circumflex artery: what is the course of the right-sided branch?

**DOI:** 10.1007/s12471-013-0515-7

**Published:** 2014-01-08

**Authors:** A. Y. Andreou

**Affiliations:** Department of Cardiology, Limassol General Hospital, Nikeas Street, Pano Polemidia, 3304 PO Box 56060, Limassol, Cyprus

Dear Editor,

I read with interest the case by Ulucay et al. [1] regarding a patient with chest discomfort in whom angiography revealed two separate left circumflex (LCx) arteries, one arising from the left main coronary artery and the other from the ostium of the right coronary artery. The patient refused to undergo computed tomography angiography (CTA) to define the course of the right-sided LCx artery and the authors speculated that the patient’s symptoms might be due to the aberrant vessel following an interarterial course between the aortic root and pulmonary trunk. This letter draws attention to the angiographic image of the aberrant LCx artery illustrating this article, which does not favour an interarterial course, and aims to prevent confusion of the readers. As seen in a left anterior oblique caudal angiographic view, the aberrant LCx artery passes just beneath the aortic root forming a caudal-posterior loop to reach its normal position in the left atrioventricular groove. Such an angiographic picture is indeed compatible with a retroaortic course that is posterior to the aorta [2]. The characteristic caudal and posterior loop formed by the retroaortic LCx artery could also have been identified in a right anterior oblique projection while during a 30° right anterior oblique ventriculography or aortography that vessel could have been seen in profile as a radiopaque ‘dot’ posteriorly and caudally to the non-coronary aortic sinus that is the aortic root sign [2,3]. To the best of my knowledge, the only case of aberrant interarterial LCx artery ever reported in the English literature is the case by Hur et al. [4] in which a 55-year-old man underwent CTA for chest pain and was found to have an interarterial right aortic sinus-connected LCx artery that crossed beneath the proximal segment of the left anterior descending artery before reaching the left atrioventricular groove; adenosine 201-thallium myocardial scintigraphy showed no perfusion defect in the territory of the ectopic vessel. Tomographic imaging techniques such as CTA are superior to conventional coronary angiography with regard to detection of aberrant coronary artery origin and proximal course, and certainly help in reaching the right diagnosis, particularly in ambiguous cases [5]. Although intussusception of the proximal ectopic segment within the aortic wall media has been shown to be a consistent feature of the interarterial course, it has also been described in a case of right aortic sinus-connected retroaortic LCx artery [6]. Hence, in such cases, particularly when the distribution of the LCx artery to the posterolateral wall is large and/or ischaemia has been documented across the LCx artery territory by a functional test or the patient presents with typical angina in the absence of significant atherosclerotic disease, CTA can be employed to investigate the possibility of an intramural retroaortic LCx artery course. The intramural segment will be narrowed relative to the distal reference extramural segment while its cross-section will be ovoid due to lateral compression [5]. However, evaluation of the pathophysiological mechanisms leading to ischaemia and quantification of their significance can only be made with the use of intravascular ultrasound scanning [6].
